# Buffalo State Insane Asylum

**Published:** 1874-02

**Authors:** James P. White


### Miscellaneous

---

Dr. James P. White made the following response to a toast to the Buffalo Insane Asylum, at a dinner given to the Common Council and City Officials at the Tifft House, recently:

Mr. Chairman and Gentlemen: At this grand re-union of our fellow citizens I am proud to have my name associated with the Buffalo State Asylum for the Insane. You will soon perceive that I cannot make a speech and I need not detain you by saying so. The only merit I will promise in my remarks is brevity. It is peculiarly gratifying to one who has lived in Buffalo for half a century and seen it expanding from a little hamlet of two thousand inhabitants to a great city of nearly two hundred thousand, to be present here this evening and by the eloquence of friends familiar with the subjects to be assured of a corresponding growth in commerce, manufactures and all the industrial pursuits important to the welfare and prosperity of a great community It is gratifying also to be assured that at the same time the religious and educational interests have received no inconsiderable attention—that the city is also being supplied with civic public buildings suitable to its requirements and adorned with beautiful parks and public grounds, that hospitals properly administered are provided for the homeless sick. In all these respects have the enterprise and benevolence of our citizens kept pace with growth in population and wealth. Finally, my friends, Buffalo is undertaking through state authority and munificence to provide for our insane. There is no subject which marks more distinctively the degree of progress in Christian civilization in any community; nothing, which proclaims "good will to men" more truly, than efforts to care and make safe provision for these bereft of reason and self-control. It is now conceded by most intelligent allenists that the insane can be best treated and cared for when collected in hospitals properly constructed and equipped. The State of New York, fully recognizing the truth enunciated by Horace Man, that "the pauper insane are the wards of the State," has upon the application of a few of our fellow citizens undertaken the erection of a suitable structure, upon lands generously donated by this city, for the reception of this class of her afflicted and unfortunate children in the Eighth Judicial District. This great work has gone on so quietly as to attract little popular attention. Yet do the Managers of this institution indulge the hope that with a suitable appropriation this winter they will be enabled to complete within the next eighteen months more than five hundred feet of frontage, with suitable kitchen and other rear buildings necessary for the reception and care of more than two hundred patients. But fellow-citizens, what it it, we are building? This institution has nothing in it of the herole or romantic. Its massive walls are to commemorate no victory of war. It is not even designed to minister to the advancement and glory of any sect or party. No my friends it is nothing of the kind. On the contrary, it springs from those common instincts and virtues of onr nature, which have received from the civilizing and Christianizing influences of our time a scope and direction unknown to the polished nations of antiquity,—to relieve suffering, both of body and mind; to rescue the helpless men and women from practices discreditable even to a barbarous age, to lead back the wandering mind out of the darkness and mazes of disease into the unclouded light of reason; to remove from many a home some stricken one whom all the arts of affection only serve to embitter rather than to console and heal; to lighten the burthen of those who have exhausted their strength and means in caring for some cherished member of the family circle; to improve this ministry to the disordered mind, by the intelligent application of medical science—such are the aims of this institution. Is it not then worthy our warmest sympathy, our deepest respect, our strongest help? To say that insanity is the loss of reason—the great prerogative of man, conveys to the dullest understanding the fact of a great calamity to the sufferer himself, but no one without a professional acquaintance with the subject can have any idea of the extent and variety of the misery inflicted by this most terrible disease—insanity. It is but a century since Virginia, to its credit, be it said, erected the first building designed exclusively for the insane. Many years elapsed before this worthy example was followed and indeed those which were built were intended as places of confinement and safety, a substitute for the gaols in which they had been previously imprisoned. Now, if the helpless insane are the wards of the State—then each and all of them are equally entitled to its care and protection. To extent them to one, and refuse them to another, to render it almost impossible for the distant counties to avail themselves of the benefits of the hospital, is to act the part of an unnatural guardian. And yet this is precisely what has been done. At this moment there are hundreds in this great state suffering every variety of hardships (in alms-houses and in unsafe homes) who are as clearly entitled to the privileges of a hospital as any of those who have been received within its walls. The State is gradually making more extensive provision for the care of her unfortunate children. But statistics conclusively show, that of the total number of insane in the State, *less than one-half* can at present be provided with suitable hospital accommodation.

No accident of fortune or birth, no measure of strength, no exercise of prudence, may be able to save us from the fate of others once as little likely to meet it as ourselves. Sad as it may seem it is nevertheless a well established fact that there is a constant parallellism between the progress of society and the increase of mental disorders; that while in aboriginal races and people insanity is comparatively unknown, it prevails in greatest frequency in nations of the highest culture and refinement. We are all, therefore, interested in urging on this goód work to a rapid conclusion.

On behalf of my colleagues and myself, to whom the State has confided the care of this great enterprise, permit me to say it shall not be our fault if it is not prosecuted with zeal and economy. May we not claim for this great humanitarian undertaking your undivided sympathy and co-operation.

